# Ejaculate investment and attractiveness in the stalk-eyed fly, *Diasemopsis meigenii*

**DOI:** 10.1002/ece3.544

**Published:** 2013-04-18

**Authors:** Elisabeth Harley, Leanna M Birge, Jennifer Small, Samuel J Tazzyman, Andrew Pomiankowski, Kevin Fowler

**Affiliations:** 1 Department of Genetics, Evolution and Environment, University College LondonDarwin Building, Gower Street, London, WC1E 6BT, UK; 2 Department of Biology, University of MarylandCollege Park, Maryland, 20742, USA; 3 CoMPLEX, University College LondonGower Street, London, WC1E 6BT, UK

**Keywords:** Mate choice, mate preference, sexual ornament, sexual selection, sperm competition, spermatophore

## Abstract

The phenotype-linked fertility hypothesis proposes that male fertility is advertised via phenotypic signals, explaining female preference for highly sexually ornamented males. An alternative view is that highly attractive males constrain their ejaculate allocation per mating so as to participate in a greater number of matings. Males are also expected to bias their ejaculate allocation to the most fecund females. We test these hypotheses in the African stalk-eyed fly, *Diasemopsis meigenii*. We ask how male ejaculate allocation strategy is influenced by male eyespan and female size. Despite large eyespan males having larger internal reproductive organs, we found no association between male eyespan and spermatophore size or sperm number, lending no support to the phenotype-linked fertility hypothesis. However, males mated for longer and transferred more sperm to large females. As female size was positively correlated with fecundity, this suggests that males gain a selective advantage by investing more in large females. Given these findings, we consider how female mate preference for large male eyespan can be adaptive despite the lack of obvious direct benefits.

## Introduction

Traditional sperm competition theory predicts that male fertilisation success following a mating is determined by the number of sperm transferred to the female (Parker 1970; Wedell et al. 2002; Pizzari and Parker 2009). Male ejaculate is likely to be limited by the costs of producing energetically expensive sperm and accessory fluids (Dewsbury 1982; Moore et al. 2004), and by the depletion of their reserves in prior matings (Nakatsuru and Kramer 1982; Preston et al. 2001). So it is expected that males will strategically adjust their ejaculates to maximize the number of matings and fertilisation success they can achieve given the limited resources they have to expend on reproduction. Males are also expected to evolve to be sensitive to a range of female characters that reflect female reproductive value, for example: age, size or mating history (Parker et al. 1999; Martin and Hosken 2002; Lupold et al. 2011). Males may also respond to demographic features that reflect the likely intensity of sperm competition, for example: phase of mating season, male dominance and the sex ratio (Wedell and Cook 1999; Bretman et al. 2010; Ingleby et al. 2010).

The strategic allocation of ejaculate by males could result in impaired fertility amongst females if males restrict the amount of sperm contained in a single ejaculate (Royer and McNeil 1993; Svensson et al. 1998). It has been frequently reported in insects that a single mating is insufficient to fertilise all of a female's eggs (Ridley 1988; Arnqvist and Nilsson 2000). Reduced female fertility is also possible if females are selected to restrict the number of matings due to fitness disadvantages associated with multiple mating (Chapman et al. 1995; Crudgington and Siva-Jothy 2000; Stutt and Siva-Jothy 2001). Under these circumstances female preference for mating with the most fertile males will be selectively advantageous (Rogers et al. 2006). Direct assessment of male fertility is unlikely. But it is possible that males advertise their reproductive quality. This idea has come to be known as the “phenotype-linked fertility” (PLF) hypothesis, and proposes that exaggerated male sexual ornaments act as indicators of male reproductive quality (Sheldon 1994; Iwasa and Pomiankowski 1999). The PLF hypothesis has been framed within the context of the handicap principle (Pizzari et al. 2004; Rogers et al. 2008), with the association between male ornament size and fertility assumed to arise because both traits are costly and so evolve similar condition-dependent expression.

The PLF hypothesis predicts that attractive males transfer larger ejaculates during mating and females gain fertility benefits through their choice of mate (Pizzari et al. 2004). However, a recent model of sperm competition, in which males vary both in the quantity of resources they can allocate to reproduction (*R*) and also in the cost of obtaining a mate (*c*), comes to a different conclusion (Tazzyman et al. 2009). The analysis models sperm competition between males as a fair raffle proportional to the amount of sperm per ejaculate. It calculates the ESS (Evolutionarily Stable Strategy) resource allocation to a mating (*s*) given the expected number of matings as *R/(c + s)*, the resources allocated to reproduction divided by the total cost per mating (i.e., the cost of obtaining a mating added to the resources allocated to a mating). Under these assumptions, males with a lower cost of obtaining a mating will value their matings less. These males are expected to mate more often and to constrain their ejaculate investment per mating, resulting in smaller ejaculates relative to those of competitors who experience a higher cost of obtaining a partner. In contrast, a male's optimal ejaculate expenditure does not vary with respect to the amount of resources allocated to reproduction (assuming cost per mating is fixed). In species where females exert mate choice using male sexual ornaments, attractive males (i.e., those with low costs of obtaining a mating) are predicted to invest less per mating. Unattractive males experiencing a high cost are expected to produce larger ejaculates (Tazzyman et al. 2009). Where males also vary in their resources allocated to reproduction, we expect no effect on ejaculate size except when these resources covary with the costs of obtaining a mate.

**Figure 1:**
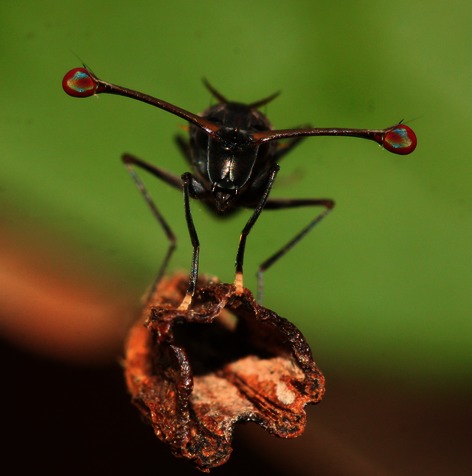
A male *Diasemopsis meigenii* stalk-eyed fly (photograph, Sam Cotton).

The PLF hypothesis predicts that ejaculate investment will be positively associated with male attractiveness, while the model of strategic allocation (Tazzyman et al. 2009) predicts a negative association. In order to test these mutually exclusive predictions, we investigated male ejaculate allocation in a model insect species, the stalk-eyed fly *Diasemopsis meigenii* (Fig. 1). These flies are characterised by the lateral displacement of their eyebulbs on long stalks. Eyespan is sexually dimorphic, with males having more widely displaced eyes than females (Baker and Wilkinson 2001) and is subject to sexual selection through female choice for large male ornamentation (Burkhardt and de la Motte 1988; Wilkinson et al. 1998; Cotton et al. 2006). Male eyespan is a highly condition-dependent trait in *D. meigenii* (Bellamy et al. 2013) and other stalk-eyed fly species (David et al. 1998; Bjorksten et al. 2001; Cotton et al. 2004). In the related stalk-eyed fly species *Teleopsis dalmanni*, eyespan is a reliable indicator of the size of male internal reproductive organs (Rogers et al. 2008; Cotton et al. 2010). A number of studies have shown that male stalk-eyed flies are sperm limited (Fry and Wilkinson 2004; Rogers et al. 2005, 2006), resulting in reduced female fertility and long-term sperm depletion (Baker et al. 2001; Rogers et al. 2006; Cotton et al. 2010; Harley et al. 2010).

We began by asking whether male eyespan acted as a signal of male reproductive investment by measuring testes and accessory gland length. We also examined whether sperm length varied with male eyespan, as there is some evidence that sperm length can influence male sperm competitiveness (Pitnick et al. 2009). Male strategic allocation of ejaculate was measured in recently mated females by calculating the size and sperm content of the spermatophore transferred. We asked how males allocate their ejaculates to larger, more fecund females by comparing the quantities of ejaculate transferred to large and small size females during a single mating. The PLF and strategic allocation hypotheses (Tazzyman et al. 2009) make contrasting predictions about how male ejaculate investment varies with male attractiveness, so we compared ejaculate allocation in a single mating amongst large and small eyespan males. Finally we briefly extend the modelling results published previously (Tazzyman et al. 2009) to include potential variation in male ejaculate investment relative to female fecundity.

## Materials and Methods

### Experimental flies

Eggs were collected from a laboratory population of *D. meigenii* and placed in groups of 13–20 into Petri dishes that contained a moist cotton pad and ∼0.4 g of ground sweet corn food medium. These conditions created a high stress larval environment, resulting in large eyespan variation between emerging flies (Rogers et al. 2006). Adult flies were sorted into single sex groups and housed in 11 L Perspex containers containing a moist cotton wool lining and ad libitum ground sweet corn food. Only sexually mature adult flies, aged 8–10 weeks post eclosion, were used in the experiments.

Eyespan (defined as the distance between the outer tips of the eyestalks) was measured to a tolerance of 0.01 mm (ImageJ v.1.46, NIH, Bethesda, MD) in adult flies (Cotton et al. 2004). As female *D. meigenii* select males based upon the length of their eyespans (Cotton et al. 2006), we divided males into large eyespan (≥7.40 mm) and small eyespan (≤7.20 mm) classes, with cut-offs either side of the mean of the distribution. In females the eyespan trait is not subject to sexual selection but is strongly correlated with body size, so females were also divided into large (≥5.40 mm) and small (≤5.20 mm) classes, again around the mean of the distribution. Other flies were discarded. The experimental flies were transferred to individual 500 mL containers lined with a moist cotton pad, and given fresh ground sweet corn food every 2–3 days.

### Reproductive investment

Reproductive investment by large and small females was measured as reproductive output. Single experimental females (large *n* = 43, small *n* = 30) were placed in containers lined with a sheet of blue paper so that eggs were visible. The number of eggs laid was counted every 2–3 days for a period of 10 days.

Male reproductive investment was measured by accessory gland and testis size. Sexually mature males were anaesthetised on ice. The accessory glands and testes were dissected out in phosphate-buffered saline (PBS) solution and transferred to a glass slide. Images of each organ were captured using a digital camera attached to a dissecting microscope at 10× magnification. The length of each pair of organs was measured by tracing a midline bisecting the length of the organ (Rogers et al. 2005). Both accessory glands and testes were measured and the mean of each pair was used in subsequent analyses (Baker et al. 2003). Data was collected from large (*n* = 23) and small (*n* = 22) eyespan males.

We measured the length of sperm stored in the testes of large (*n* = 15) and small (*n* = 13) eyespan males. Male testes were dissected out as described above, and gently ruptured to release the sperm bundles. We measured the lengths of four mature sperm bundles and used the mean of each quartet in the analyses.

### Ejaculate investment per mating

Two separate experiments were carried out to investigate strategic allocation of ejaculate. In the first experiment, we tested whether variation in female size results in different size or quality of ejaculate transferred. Males (all large eyespan) were mated once to either a large (*n* = 119) or a small (*n* = 91) female. In the second experiment, we tested whether variation in male eyespan results in different size or quality of ejaculate. Large (*n* = 110) or small (*n* = 104) eyespan males were mated singly to large virgin females.

In both experiments, matings were conducted by transferring a male into a female's container at dawn (∼0900 h). The time to copulation and the duration of the copulation were recorded to the nearest second, to establish whether they correlated with size or quality of ejaculate. A mating was defined as genital engagement for longer than 150 sec, the length of time known to be needed for sperm transfer to take place (E. Harley, unpubl. data). Females sometimes rejected mating attempts by males (Cotton et al. 2006) or the male disengaged after <150 sec (typically within 20 sec). In either case, this was recorded as a rejection. The male was allowed to make further mating attempts for up to half an hour. If a mating had still not happened, the female (male) was replaced in the male (female) eyespan (size) variation experiment, and the procedure repeated. The individual replaced was drawn from the same size class. If there was still no successful mating after a further half hour, that individual was removed from the study. All males and females used were sexually mature virgins and were only used once.

Immediately following the mating, the female was anaesthetised on ice and her reproductive tract dissected out into 25% glycerol/PBS (pH 7.2). A coverslip was placed gently over the reproductive tract and the spermatophore was viewed using a DIC-equipped binocular microscope. Photographs were taken at 400× magnification using a Nikon CoolPix (Tokyo, Japan) digital camera. Male *D. meigenii* transfer sperm to females in a spermatophore envelope of accessory proteins (Kotrba 1996). Spermatophore area was measured to the nearest 0.0001 mm^2^ (ImageJ v1.46, NIH). The number of sperm contained in a spermatophore is impossible to quantify as the sperm are tightly coiled into a dense mass. So as a proxy we calculated the area of the spermatophore occupied by sperm (Appendix S1).

### Statistical analyses

We used *F*-tests or General Linear Models (GLMs) to evaluate the effect of female size (large and small) upon: fecundity, area of the spermatophore transferred, absolute area of sperm in the spermatophore and relative size of sperm transferred (controlling for spermatophore area). Tests for whether sperm content and spermatophore area were positively correlated were carried out to see if it was appropriate to use spermatophore area as a covariate in GLMs testing for variation in sperm content. We tested whether the time to copulation (after log transformation to control for the non-normal distribution of this variable) or copulation duration differed between large or small females, or predicted spermatophore characteristics. These tests were repeated to evaluate the effect of male eyespan (large and small) on the same variables (excluding fecundity), and additionally including accessory gland, testes, and sperm bundle length.

We tested for an association between occurrence of rejection (measured as occurring or not) and time to copulation, copulation duration and spermatophore characteristics. A *χ*^2^ contingency analysis was used to determine the effect of male eyespan and female size upon the occurrence of rejection. All analyses were performed using JMP statistical software (SAS, Cary, NC).

### Model of optimal sperm allocation

We adapted a prior model (Tazzyman et al. 2009) to consider variation in male ejaculate investment relative to female fecundity. Briefly, males have a quantity of resources *R* to allocate to mating. They are subject to a cost *c* which describes the quantity of resources they expend in order to obtain each mating. Their strategy then consists of the quantity *s* of resources that they allocate to each mating. Since the number of matings they can afford will be *n*(*s*|*R*,*c*) = *R*/(*c* + *s*), the smaller the value of *s* the more matings a male can afford. However, the success per mating is a function *v*(*s*) which increases with *s*. For details of the function see Tazzyman et al. (2009).

In the original model (Tazzyman et al. 2009) all females were assumed to be identical. Here we adapt this framework by assuming there are two types of female which differ in fecundity. Normal females, which make up a proportion *q* of the population of females, have fecundity 1. Fecund females, which make up a proportion 1 – *q* of the population of females, have fecundity 1 + *h*. We assume that the two types of female are identical in mating preference. Males are assumed to be able to detect the difference in female fecundity, and to adopt independent ejaculate allocation strategies for each type of female (*s*_1_ for normal females and *s*_2_ for fecund females). Using the techniques set out in (Tazzyman et al. 2009), we derive the ESS strategies *s*_1_ and *s*_2_.

## Results

### Reproductive investment

**Figure 2:**
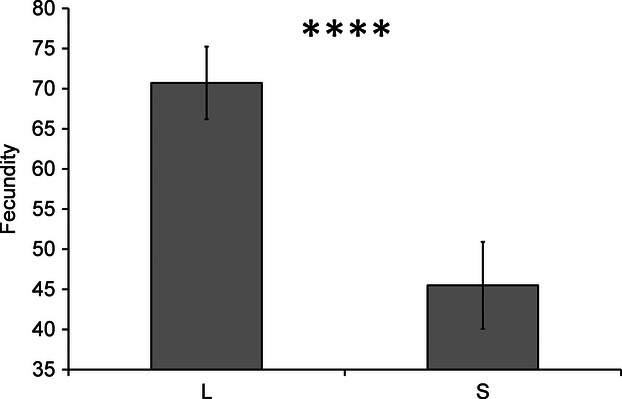
Effects of female eyespan class (L and S) upon mean female fecundity over a 10 day period. Error bars show ± SEM. Degree of significance is shown using asterisks (****P < 0.0001).

**Figure 3:**
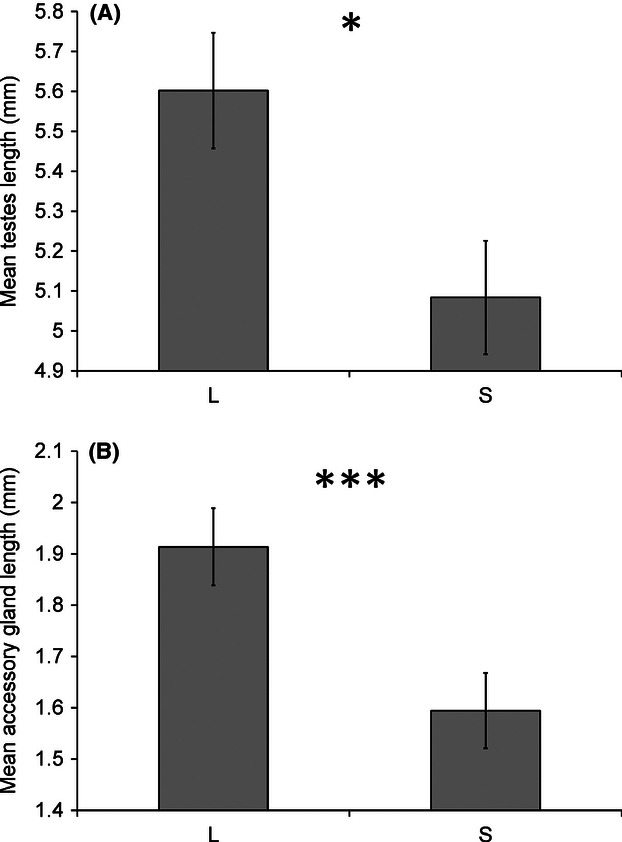
The relationship between (A) male eyespan class (L and S) and mean testis length (mm), and (B) male eyespan class (L and S) and mean accessory gland length (mm). Error bars show ± SEM. Degree of significance is shown using asterisks (*P < 0.05; ***P < 0.001).

To assess whether large females have higher reproductive value to males, we measured female fecundity. Large females laid significantly more eggs during a 10 day period than small females (*F*_1,71_ = 12.7725, *P =* 0.0006; Fig. 2). To assess whether large eyespan males have higher reproductive capacity, we measured their testes and accessory glands. Large eyespan males had significantly larger testes (*F*_1,43_ = 6.5223, *P* = 0.0143; Fig. 3A) and larger accessory glands (*F*_1,37_ = 9.2252, *P* = 0.0041; Fig. 3B) than small eyespan males. Sperm bundle length was consistent across the two groups of males, with no effect of male eyespan size class observed (L males: 1.8922 ± 0.0217 mm, S males: 1.8715 ± 0.0233 mm, *F*_1,26_ = 0.4219, *P* = 0.5217).

### Effect of female size on male ejaculate investment

**Figure 4:**
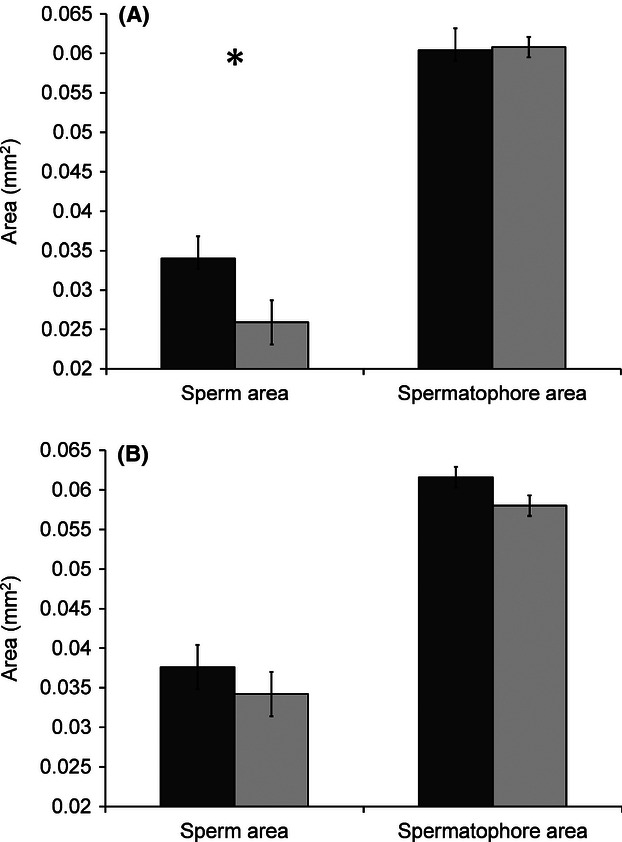
Effects of female size (A) and male eyespan (B) variation (large eyespan: dark bars; small eyespan: light bars) upon spermatophore area (mm^2^) and absolute sperm content (mm^2^). Error bars show ± SEM. Significant differences between eyespan classes are shown with an asterisk (*P < 0.05).

Males did not vary the size of spermatophore transferred during mating in relation to female size (*F*_1,158_ = 0.0422, *P* = 0.8375; Fig. 4A). However, males transferred spermatophores with greater absolute sperm content when mating with large females (*F*_1,158_ = 5.4511, *P* = 0.0208; Fig. 4A). Spermatophore area and sperm content were highly positively correlated (*r =* 0.489, *n* = 160, *P* < 0.0001), so we repeated this test with spermatophore area as a covariate, and still found that sperm content differed between the female size classes, with large females receiving relatively more sperm (L females: 0.0342 ± 0.0024 mm^2^, S females: 0.0247 ± 0.0024 mm^2^; *F*_1,157_ = 7.8705, *P* = 0.0057).

**Figure 5:**
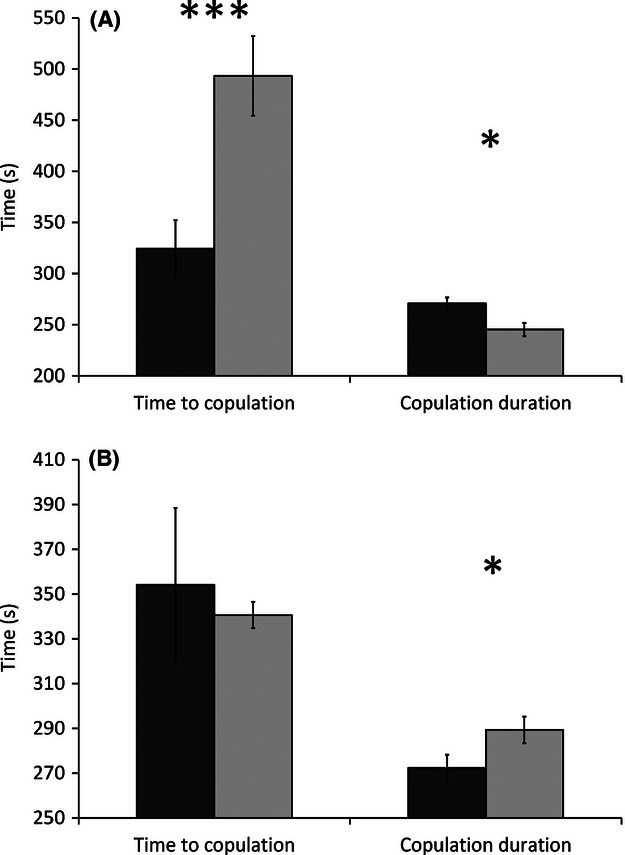
The effect of female size (A) and male eyespan (B) (large eyespan class: dark bars; small eyespan class: light bars) upon time to copulation and copulation duration. Error bars show ± SEM. Significant differences between eyespan classes are shown with asterisks (*P < 0.05, ***P < 0.001).

Males took less time to start copulating with large females (*χ*^2^_1,208_ = 14.7039, *P* = 0.0002; Fig. 5A) and copulated for longer with large females (*F*_1,208_ = 5.4625, *P* = 0.0204; Fig. 5A). Neither time to copulation nor copulation duration had a significant effect upon spermatophore area (time to copulation *χ*^2^_1,158_ = 0.4710, *P* = 0.4935; copulation duration: *F*_1,158_ = 0.2277, *P* = 0.6339), absolute sperm content (time to copulation: *F*_1,158_ = 0.0910, *P* = 0.7633; copulation duration: *F*_1,158_ = 0.0165, *P* = 0.8981) or relative sperm content (time to copulation: *F*_1,157_ = 0.0015, *P* = 0.9697; copulation duration: *F*_1,157_ = 0.0142, *P* = 0.9049).

### Effect of male eyespan on male ejaculate investment

Male eyespan had no significant effect upon spermatophore area (*F*_1,158_ = 3.7101, *P* = 0.0559; Fig. 4B). As this was border-line significant, we examined the distributions for outliers, but found that their exclusion reduced the difference (*F*_1,1556_ = 1.6715, *P* = 0.1980) as they all belonged to small eyespan males (*n* = 3). Neither did male eyespan influence absolute sperm content (*F*_1,158_ = 0.7355, *P* = 0.3924; Fig. 4B). As in the female size variation experiment, spermatophore area and sperm content were highly positively correlated (*r =* 0.461, *n* = 160, *P* < 0.0001). After taking account of spermatophore area, there was still no difference in relative sperm content associated with male eyespan (*F*_1,157_ = 0.0006, *P* = 0.9799).

Male eyespan had no significant effect upon time to copulation (*χ*^2^_1,212_ = 0.5341, *P* = 0.4657; Fig. 5B), but it did affect copulation duration as small eyespan males copulated for longer than large eyespan males (*F*_1,212_ = 4.0814, *P* = 0.0446; Fig. 5B). Neither time to copulation or copulation duration had a significant effect upon spermatophore area (time to copulation: *F*_1,158_ = 0.0049, *P* = 0.9443; copulation duration: *F*_1,158_ = 0.0026, *P* = 0.9590), absolute sperm content (time to copulation: *F*_1,158_ = 0.2758, *P* = 0.6002; copulation duration: *F*_1,158_ = 0.1161, *P* = 0.6887) or relative sperm content (time to copulation: *F*_1,157_ = 0.3924, *P* = 0.5319; copulation duration: *F*_1,157_ = 0.1800, *P* = 0.6719).

### Female rejection and copulation failure

Females rejected males in 16% of pairings, before eventually accepting them (*n* = 68 out of 424). As expected, rejection significantly increased the time to copulation, both in the female size (no rejection: 384.52 ± 28.51 sec; rejection: 555.51 ± 59.54 sec; *F*_1,191_ = 3.983, *P* = 0.0474) and male eyespan experiments (no rejection: 287.54 ± 25.90 sec; rejection: 557.50 ± 59.34 sec; *F*_1,198_ = 13.078, *P* < 0.0004). But there was no evidence that female size affected rejection rates in the female size experiment (*χ*^2^_1,193_ = 0.224, *P* = 0.6362), or that male eyespan affected rejection rates in the male eyespan experiment (*χ*^2^_1,200_ = 0.345, *P* = 0.5569). Nor were there any associations of rejection with copulation duration, spermatophore area, absolute sperm content or relative sperm content in either the female size or the male eyespan experiments (Table 1).

**Table 1:** Correlates of female rejection in the female size and male eyespan experiments

	Rejected males, mean ± SE (*n*)	Accepted males, mean ± SE (*n*)	
Female size experiment			
Copulation duration	262.11 ± 10.34 sec (36)	258.57 ± 4.95 sec (157)	*F*_1,191_ = 0.0952, *P* = 0.7580
Spermatophore area	0.057 ± 0.002 mm^2^ (27)	0.061 ± 0.001 mm^2^ (122)	*F*_1,147_ = 2.9188, *P* = 0.0897
Absolute sperm content	0.027 ± 0.0048 mm^2^ (27)	0.029 ± 0.002 mm^2^ (122)	*F*_1,147_ = 0.1386, *P* = 0.7102
Relative sperm content	0.031 ± 0.004 mm^2^ (27)	0.029 ± 0.002 mm^2^ (122)	*F*_1,146_ = 0.2215, *P* = 0.6386
Male eyespan experiment			
Copulation duration	272.56 ± 10.59 sec (32)	279.43 ± 4.62 sec (168)	*F*_1,198_ = 0.3540, *P* = 0.5526
Spermatophore area	0.059 ± 0.003 mm^2^ (18)	0.059 ± 0.001 mm^2^ (131)	*F*_1,147_ = 0.0312, *P* = 0.8600
Absolute sperm content	0.032 ± 0.006 mm^2^ (18)	0.036 ± 0.002 mm^2^ (131)	*F*_1,147_ = 0.4603, *P* = 0.4985
Relative sperm content	0.032 ± 0.005 mm^2^ (18)	0.036 ± 0.002 mm^2^ (131)	*F*_1,146_ = 0.4550, *P* = 0.5010

Almost 25% of matings (*n* = 104 out of 424) did not result in successful spermatophore transfer. In these cases the spermatophore was either misshapen and empty, or completely absent. We found no effect of female size (L females: 27 failures, S females: 23 failures; *χ*^2^_1,210_ = 0.2240, *P* = 0.6214) or male eyespan (L males: 30 failures, S males: 24 failures; *χ*^2^_1,214_ = 0.4990, *P* = 0.4800) upon the occurrence of copulation failure.

### Model of optimal sperm allocation

Using an evolutionary game theory approach it can be shown (see Appendix S2) that for all males, the ESS strategy (*s*_1_*, *s*_2_*) has the feature that *s*_2_* = (1 + *h*)*s*_1_*. Since fecund females are (1 + *h*) times more fecund than normal females, males value matings with them as being (1 + *h*) times more valuable. Thus at the ESS, males invest (1 + *h*) times ejaculate per mating. As shown by the original model (Tazzyman et al. 2009), at the ESS, ejaculate investment increases with the cost of obtaining a mating (*c*) (i.e., decreases with male attractiveness). This investment is independent of the quantity of resources *R* that a male has to allocate to reproduction.

## Discussion

Ejaculate limitation places a significant selective pressure on males to invest their reproductive resources strategically, typically directing more ejaculate to females with higher reproductive value (reviewed in Wedell et al. 2002). In *D. meigenii*, we found that female size was strongly positively correlated with female fecundity. So our expectation was that males should direct more sperm to larger females. To formalize this hypothesis, we added variation in female fecundity to a model of sperm allocation that already incorporates sperm competition (Tazzyman et al. 2009), and showed that males should allocate higher quantities of sperm to more fecund females. In line with several other studies in insects and other species (Wedell et al. 2002), our experiments largely confirm this prediction. We found that males allocated more sperm to large females. We found that sperm content was correlated with spermatophore size, so we estimated the relative sperm content transferred and found that this too was positively associated with female size. This means that for a given spermatophore size, more sperm were transferred to large females. However, there was no difference in spermatophore size transferred to large and small females.

We also investigated whether variation in male sexual attractiveness, as determined by male eyespan, altered male ejaculate allocation. There was no difference in spermatophore size, the amount of sperm transferred, or in the relative amount of sperm transferred, between large and small eyespan males. The PLF hypothesis (Trivers 1972; Sheldon 1994) suggests that females prefer to mate with males bearing larger sexual ornaments as these males are capable of investing more resources into each mating, resulting in increased fertility benefits for females. Our results do not support this prediction of the PLF hypothesis.

The PLF hypothesis has been formally investigated in a sperm competition model in which males varied in attractiveness (the costs of gaining a mating) and in the resources they have to allocate to reproduction (Tazzyman et al. 2009). This theoretical analysis also failed to support the PLF hypothesis. The model found that attractive males constrain their investment per mating as they have more mating opportunities, predicting that attractive males produce smaller ejaculates or fewer numbers of sperm per mating (Tazzyman et al. 2009). When males differed in the resources committed to reproduction, they were not found to alter ejaculate allocation per mating. Instead, males with greater resources were predicted to mate more often (Tazzyman et al. 2009). These observations are relevant here as we found that male eyespan was a predictor of both of testis and accessory gland size in *D. meigenii*. Consequently, in *D. meigenii,* male attractiveness and resources allocated to reproduction are positively correlated. A similar finding was reported in *T. dalmanni,* another stalk-eyed species (Rogers et al. 2008). Though findings like these have been interpreted as supporting the PLF hypothesis (e.g., Pizzari et al. 2004; Rogers et al. 2008; Small 2009) this is not a reasonable deduction as reproductive organ size may scale with attractiveness in order to allow more attractive males to successfully mate more often rather than to increase their ejaculate size per mating. As a result, the number of matings and ejaculate size are likely to be coupled to condition, as has been demonstrated in *T. dalmanni* (Rogers et al. 2008).

Male eyespan did significantly influence copulation duration: small eyespan males mated for longer than large eyespan males. This could be indicative of increased investment per mating by unattractive males, as predicted by Tazzyman et al. (2009). However, we found that copulation duration was not significantly associated with either spermatophore size or sperm content. This suggests that variation in male copulation duration is associated with factors beyond simple ejaculate transfer. One possibility is that large eyespan males are subject to selection to reduce the amount of time they spend per mating, so as to exploit other mating opportunities. Our observations suggest that unattractive small males benefit in some way from longer copulations. Perhaps longer copulation duration ensures that a greater proportion of sperm are transferred to storage. Males may engage in longer copulations with large females, as we observed, for similar reasons. This possibility will be worth further investigation.

**Figure 6:**
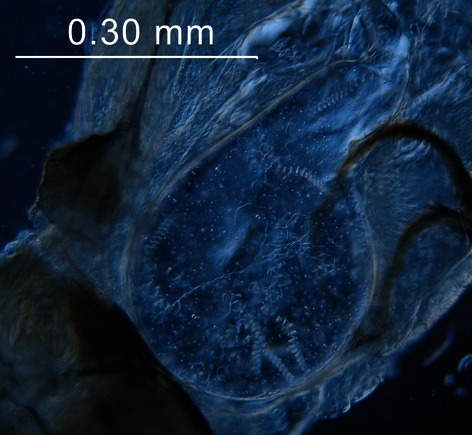
The spermatophore of *Diasemopsis meigenii*, photographed in the female reproductive tract, at 200× magnification.

We measured quantity of sperm as the proportional area within the spermatophore that contained sperm pixels, rather than the more commonly reported value of sperm number. Consequently we cannot be certain that the observed differences in male ejaculate allocation strategy are due to differences in sperm number. The mass of sperm in the spermatophore was typically highly tangled and overlaid (see Fig. 6), so we could only estimate the area of the spermatophore in which sperm were present. It is possible that sperm length varied across female size classes, and this contributed to the differences observed. However, our examination of sperm bundle length does not suggest that there is much variation in the length of sperm between large and small eyespan males. It seems far more plausible that the differences observed are due to sperm number. As well as sperm, males transferred accessory gland proteins in the spermatophore. The role of these ejaculate proteins (aside from spermatophore formation) remains poorly understood in stalk-eyed flies (Kotrba 1996). Accessory gland proteins are likely to have important post-copulatory roles in stalk-eyed flies, as has been observed in *D. melanogaster* (Chapman 2001). Quantification of accessory gland proteins would be useful in order to determine whether spermatophore protein content covaries positively with sperm number. The proteins contained in an ejaculate are also likely to be an important component of male ejaculate allocation strategy.

Our results do not support the PLF hypothesis, but neither do they completely support the alternative hypothesis proposed by Tazzyman et al. (2009). This model predicts that attractive males should invest less per mating, whereas there should be no effect of variation in male resources on sperm allocation (even if sperm allocation co-varies with male attractiveness). Eyespan is known to increase male attractiveness in *D. meigenii*, but we found no evidence that large eyespan attractive males reduced their ejaculate investment as predicted (Tazzyman et al. 2009). This finding suggests that the model (Tazzyman et al. 2009) does not fully capture the selective pressures operating on sperm allocation strategy. An important possibility to consider is the temporal clumping of mating. In many species, mating is limited to particular periods in the day. This is likely to cause differences in the mating schedule with respect to attractiveness. We expect that attractive males ought to have evolved to cope with multiple mating during the period(s) in the day when matings occur, and with a higher overall rate of mating. In contrast, less attractive males may expect a more sporadic pattern of mating with a far lower likelihood of multiple mating in any mating period. These features should result in differences in how quickly individuals suffer from depletion of their ejaculate reserves with knock-on effects on the number and size of ejaculates that can be produced (Wedell et al. 2002). In the future, these issues need to be investigated both theoretically and empirically.

## Supporting Information

**Appendix S1.** Calculation of sperm content using pixel counting.

**Appendix S2.** Model of optimal sperm allocation.
