# Supplementary material for: Ejaculate investment and attractiveness in the stalk-eyed fly, Diasemopsis meigenii
Source: Ecol Evol. 2013 Apr 18;3(6):1529–38. doi: 10.1002/ece3.544 (PMC3686189; doi:10.1002/ece3.544)
Supplement: Supplementary file 1 [file ece30003-1529-SD1.pdf]

Appendix 1: Calculation of sperm content using pixel counting

Sperm overlap with one another within the spermatophore, making sperm number very difficult to quantify. Therefore a programme was designed and written by Brian K. Birge (NASA) for estimating the percentage area of a spermatophore occupied by sperm using the numerical computing environment Matlab.

The programme analyses digital images of spermatophores and counts pixels based upon brightness. Sperm are distinguishable as they are typically paler than the surrounding images. When making measurements we equalised the image contrast and converted the colours to grey scale. The boundary of the spermatophore excluding the tubular neck was manually marked. An arbitrary threshold was then chosen which on visual inspection classified sperm and non-sperm accurately. If the sperm were dispersed through a spermatophore then we were able to define a sub-region of the spermatophore within which to count sperm pixels. This improved accuracy by avoiding the mis-counting of paler pixels as sperm in regions of the spermatophore where visual inspection revealed that there were none.

The programme returned the percentage of the total spermatophore area that was occupied by sperm pixels. Using our independent measurements of sperm content (see Materials and Methods) we converted these percentages into absolute measurements, using the following equation,

$$sperm\ content = \frac{programme\ output\ (\%\ area) \times spermatophore\ area}{100}$$

Appendix 2: Model of optimal sperm allocation

Background

Suppose there are two types of females in the population: a proportion q of normal females, and a remaining proportion $1 - q$ of super-fecund females. The normal females have fecundity 1, while the super-fecund females have fecundity $1 + h$.

Strategy

A male's strategy is a vector $\mathbf{s} = (s_1, s_s)$ describing the quantity of resources he will assign to a mating with female types 1 and 2. Since both s_1 and s_2 must be positive real numbers, we can characterise a male's strategy as being $\mathbf{t} = (s_1, x)$, where x is the (positive real) coefficient such that $s_2 = x\, s_1$.

Males have two key parameters, their quantity of resources allocated to reproduction R, and the resource cost that they must pay to obtain a mating, c. A male's strategy will be conditional upon these parameters, so that it will be a function $\mathbf{s}[R,c]$. The optimal strategy for a male depends upon what the rest of the population is doing. We denote the mean population strategy as $\bar{\mathbf{s}} = (\bar{s}_1, y\bar{s}_1)$, where y is the (positive real) coefficient such that $y\bar{s}_1 = \bar{s}_2$.

Fitness function

The fitness function for a male with resources R, and cost c (hereafter referred to as an (R,c)-male) playing strategy $\mathbf{s}[R,c]$ is defined as,

$$W[\mathbf{s},R,c|\overline{\mathbf{s}}]=n[\mathbf{s},R,c]\cdot v[\mathbf{s},R,c|\overline{\mathbf{s}}],$$

where $n[\mathbf{s},R,c]$ is the expected number of matings, and $v[\mathbf{s},R,c|\overline{\mathbf{s}}]$ is the expected success per mating. If we denote the partial derivatives of W with respect to s_1 and to s_2 by W_1 and W_2 respectively, then a strategy $\mathbf{s}$ can only be a best reply to a population mean strategy $\overline{\mathbf{s}}$ if,

$$W_1[\mathbf{s},R,c|\overline{\mathbf{s}}]=W_2[\mathbf{s},R,c|\overline{\mathbf{s}}]=0,$$

and we also have that for i = 1,2,

$$W_i[\mathbf{s},R,c|\overline{\mathbf{s}}]=n_i[\mathbf{s},R,c]\cdot v[\mathbf{s},R,c|\overline{\mathbf{s}}]+n[\mathbf{s},R,c]\cdot v_i[\mathbf{s},R,c|\overline{\mathbf{s}}]$$

where n_i and v_i refer to partial derivatives with respect to i.

Number of matings

We define the expected number of matings by,

$$n[\mathbf{s},R,c]=\frac{R}{c+qs_1+(1-q)s_2}.$$

This is the ratio of the quantity of resources that the male possesses to the average quantity of resources he uses per mating. This is an approximation to the expected number of matings given **s**, *R*, and *c*, but simulations showed it to be a reasonable approximation under a range of reasonable parameter values (results not shown).

We then have,

$$n_1[\mathbf{s},R,c]=\frac{-qR}{(c+qs_1+(1-q)s_2)^2}\ ,$$

$$n_2[\mathbf{s},R,c]=\frac{-(1-q)R}{(c+qs_1+(1-q)s_2)^2}\ ,$$

(1)

so that $n_2/n_1=(1-q)/q$.

Success per mating

The expected success per mating is then defined as

$$v[\mathbf{s},R,c|\bar{\mathbf{s}}]=\sum_{k=0}^{\infty}\frac{\bar{n}^k e^{-\bar{n}}}{k!}\left(\frac{qs_1}{s_1+k\bar{s}_1}+\frac{(1-q)(1+h)s_2}{s_2+k\bar{s}_2}\right).$$

The term in brackets in the summand represents the expected success from mating with a randomly chosen female who also mates with *k* other males. With a probability *q* this female is a normal female, and the focal male invests s_1 resources and receives expected success $s_1/(s_1+k\bar{s}_1)$, which is the proportion of total sperm the

female receives that belongs to him. With a probability (1 – q), the female is super-fecund, and the focal male invests s_2 resources and receives expected success $((1+h)s_2)/(s_2+k\overline{s}_2)$, which is the proportion of total sperm the female receives that belongs to him multiplied by her fecundity coefficient 1 + h.

We then have that,

$$v_1[s_1,R,c|\overline{s}_1]=\sum_{k=0}^{\infty}\frac{\overline{n}^k e^{-\overline{n}}}{k!}\left(\frac{qk\overline{s}_1}{\left(s_1+k\overline{s}_1\right)^2}\right),$$

$$\begin{aligned}v_2[s_2,R,c|\overline{s}_2]&=\sum_{k=0}^{\infty}\frac{\overline{n}^k e^{-\overline{n}}}{k!}\left(\frac{(1-q)(1+h)k\overline{s}_2}{\left(s_2+k\overline{s}_2\right)^2}\right)\\&=\sum_{k=0}^{\infty}\frac{\overline{n}^k e^{-\overline{n}}}{k!}\left(\frac{(1-q)(1+h)ky\overline{s}_1}{\left(xs_1+ky\overline{s}_1\right)^2}\right)\\&=\frac{1-q}{q}\frac{1+h}{y}v_1\left[\left(x/y\right)s_1,R,c|\overline{s}_1\right],\end{aligned}\qquad(2)$$

Analysis

Suppose there exists an evolutionary stable strategy (ESS) $\mathbf{s}^*=(s_1^*,s_2^*)$ for each combination (R,c), and suppose the population is playing this strategy. Then the mean population strategy will be $\overline{\mathbf{s}}^*=(\overline{s}_1^*,\overline{s}_2^*)=(\overline{s}_1^*,y^*\overline{s}_1^*)$. Since it is an ESS, this strategy must be a best reply to itself, i.e. for all (R,c) combinations,

$$W_1\left[\mathbf{s}^*[R,c],R,c\middle|\overline{\mathbf{s}}^*\right]=W_2\left[\mathbf{s}^*[R,c],R,c\middle|\overline{\mathbf{s}}^*\right]=0.$$

Since $W_1 = 0$,

$$n_1[\mathbf{s}^*, R, c] \cdot v[\mathbf{s}^*, R, c | \overline{\mathbf{s}}^*] + n[\mathbf{s}^*, R, c] \cdot v_1[s_1^*, R, c | \overline{s}_1^*] = 0 ,$$

which gives,

$$v[\mathbf{s}^*, R, c | \overline{\mathbf{s}}^*] = \frac{-n[\mathbf{s}^*, R, c] \cdot v_1[s_1^*, R, c | \overline{s}_1^*]}{n_1[\mathbf{s}^*, R, c]} .$$

(3)

Also,

$$n_2[\mathbf{s}^*, R, c] \cdot v[\mathbf{s}^*, R, c | \overline{\mathbf{s}}^*] + n[\mathbf{s}^*, R, c] \cdot v_2[s_2^*, R, c | \overline{s}_2^*] = 0 ,$$

which means, from (2) and (3), and then (1),

$$\frac{1+h}{y^*} = \frac{v_1[s_1^*, R, c | \overline{s}_1^*]}{v_1\left[\left(x^*/y^*\right)s_1^*, R, c | \overline{s}_1^*\right]} .$$

(4)

Since v_1 is a sum of positive decreasing functions of s_1, it is itself positive and decreasing in s_1. Equation (4) shows that if $y^* > (1+h)$, then $x^* < y^*$ for all (R,c). But this is a contradiction because then the mean population strategy for the population is not equal to $y^* \overline{s}_2^*$. A similar contradiction occurs if $y^* < (1+h)$. Therefore if an ESS

exists it must have $x^* = y^* = 1 + h$ for all (*R*,*c*), which implies that all males must invest (1 + *h*) times as much sperm in super-fecund females as they do in normal females.

It can be proven that the ESS strategy exists and is always of this form, and also that it will always increase in *c* and be independent of *R*, but these details have been discussed previously in (Tazzyman et al*.* 2009) and are not necessary here.
